# The Role of Pre-filled Procedure-Specific Consent Forms in Emergency General Surgery: A Quality Improvement Study

**DOI:** 10.7759/cureus.88660

**Published:** 2025-07-24

**Authors:** Claudia Zizzo, Maria Henriques, James Wooding, Andrew Kennedy-Dalby

**Affiliations:** 1 Emergency, Great Western Hospital, Swindon, GBR; 2 Medicine and Surgery, Whiston Hospital, Whiston, GBR; 3 Surgery, Whiston Hospital, Whiston, GBR

**Keywords:** consenting in surgery, informed consent, patient education, quality improvement research, surgical consent forms

## Abstract

Introduction

Informed consent is a crucial component of surgical practice, ensuring that patients understand the risks and benefits associated with procedures. However, handwritten consent forms are often incomplete, illegible, and inconsistent. This quality improvement project aimed to review the informed consent process for emergency laparoscopic cholecystectomy and appendicectomy at a district general hospital in the United Kingdom and subsequently introduce pre-filled, procedure-specific consent forms for these procedures.

Methods

A retrospective review of 20 handwritten consent forms compared the documented risks to a standardized set (SS) of risks developed from multiple resources. This demonstrated the inadequate documentation of the risks of handwritten consent forms. In response to this, two pre-filled consent forms were developed, which incorporated the SS of risks. Layman’s explanations were included, and space for additional risks was provided to cater to patient-specific needs. These new forms aimed to enhance patient-surgeon communication, facilitate patient understanding, and improve documentation. Post-intervention, a second retrospective analysis of 74 consent forms was then performed.

Results

Pre-intervention, a mean of 64.00% (n = 10) of risks was mentioned for appendicectomy and 55.79% (n = 10) for cholecystectomy. The most commonly documented risks were bleeding, bruising, and infection, while less frequent risks such as infertility were often omitted. Post-intervention, the mean documentation of risks increased to a mean of 81.82% (n = 45) for appendicectomy and 79.69% (n = 29) for cholecystectomy.

Conclusion

This quality improvement project demonstrates that pre-filled, procedure-specific consent forms are a valuable tool in improving the consenting process, acting as aids for structured discussion of risks, improving risk documentation, and reducing human errors in documentation. As such, a set of recommendations was developed for the future development of consent forms. Additionally, these consent forms may aid the transition to digital-based systems, helping reduce paper waste. Future recommendations also include the implementation of teaching sessions on informed consent to improve the consent process.

## Introduction

Informed consent is one of the most important ethico-legal responsibilities of healthcare practitioners. It ties into the main ethical pillars of medicine, enabling patients to exercise autonomy by thoroughly weighing the benefits and risks of the therapeutic interventions being proposed. This is especially significant in the surgical field. 

Multiple studies have analyzed the process of informed consent in surgery and found that communication and documentation during patient consent discussions are often lacking [1-3]. This not only affects patient care and satisfaction but also exposes surgeons and physicians to potential litigation [2]. When reviewing handwritten consent forms, studies have shown that poor handwriting and illegibility hinder the documentation process [3]. Pre-filled, procedure-specific consent forms have been trialed in various departments and hospitals, demonstrating some benefit to the overall consent process [2,3].

This quality improvement project aimed to improve the documentation of risks on consent forms for laparoscopic cholecystectomy and appendicectomy at a UK district general hospital. The intervention made was the implementation of pre-filled, procedure-specific consent forms. A retrospective analysis was conducted to evaluate the number of documented risks, which demonstrated inconsistent and incomplete documentation. In response, pre-filled emergency surgery consent forms were created for both laparoscopic cholecystectomy and appendicectomy. The primary aim was to improve the documentation of risks and minimize human factors such as illegibility. Furthermore, the forms were designed to help facilitate patient-surgeon discussions and improve risk comprehension while also being translatable to future digital systems, continuing sustainability practices within the trust. 

Literature review

Informed consent may be defined as:

*“A process in which patients are given important information, including possible risks and benefits, about a medical procedure or treatment, genetic testing, or a clinical trial. This is to help them decide if they want to be treated, tested, or take part in the trial. Patients are also given any new information that might affect their decision to continue. Also called consent process.”* - NIH Dictionary of Cancer Terms [4]

Despite informed consent being one of the key ethical pillars in medicine and surgery, it has been noted across multiple studies that healthcare providers fall short in this process [1]. Handwritten consent forms have been noted to have multiple omissions of benefits or risks and often include little detail [1-3]. They are also further complicated by human factors such as illegible handwriting [1,3]. Variations in what risks were consented for were also noted, the risks mentioned by consultants differing from those of doctors in training [5]. Pre-filled consent forms or stickers have been used previously as a way to help improve the consenting process, by improving the documentation of risks, including consent forms for laparoscopic cholecystectomy, one of our target procedures [6]. It has been noted that pre-filled consent forms when used alongside teaching sessions on consenting are especially beneficial [2,7]. It has also been shown that implementing pre-filled consent forms helps reduce errors and omissions and improves factors such as legibility [3,8]. Furthermore, it was demonstrated that using pre-filled consent forms also improved patient recollection of risks and provided a good framework for the consenting discussion [6,9,10]. However, pre-filled consent forms may not encompass the individual needs of patients undergoing surgery. As such, they should not be relied upon solely for true informed consent, which is an individual process [11]. This issue was deemed reducible when consent forms were able to be customized to the patient [12]. 

Additionally, handwritten consent forms are associated with increased cost. This is due to the cost of printing and scanning such forms. One study estimated that paper-based consent forms cost more than £0.90 per form compared to their digital counterparts [13]. The shift to a digital-based system also aids NHS trusts in their mission to develop more sustainable practices, reducing paper waste, in line with the NHS Net Zero scheme [14]. As such, by designing consent forms that are easily translatable to a digital format, the first steps toward a fully paperless consenting system can be undertaken.

This quality improvement project was presented at the Association of Surgeons in Training (ASiT)'s 49th Annual Surgical Conference, and the abstract submitted for the conference will be published in the BJS publication.

## Materials and methods

This quality improvement study was performed in two cycles: evaluating current practices and subsequently measuring any improvements made in the documentation of risks after implementing pre-filled, procedure-specific consent forms.

Cycle 1 involved baseline data collection and the development of initial consent forms. First, a standardized set (SS) of risks was created for both laparoscopic cholecystectomy and appendicectomy. These would act as the "gold standard" when compared to the documented risks on handwritten consent forms. Three resources were used, two of which are free for public use: (i) NHS Patient Information [15,16] and (ii) Cambridge University Hospitals Patient Information [17,18]. The third resource was (iii) BMJ Best Practice, which is freely available to NHS staff with a subscription through their institution or via a personal subscription, and is a non-commercial use service [19,20]. The SS described 14 risks for laparoscopic appendicectomy and 17 risks for laparoscopic cholecystectomy. 

A retrospective analysis was performed spanning four months. Data from 20 consent forms were analyzed. Each consent form was treated as a single unit, with the number of risks documented recorded per form. As such, the sample size (n) reflects the number of consent forms reviewed.

The risks documented on the consent forms were then analyzed in two ways: (i) the frequency each individual risk was mentioned across all consent forms and (ii) the number of risks documented per consent form, expressed as a percentage of the total risks in the SS for that procedure.

Following this, draft versions of the procedure-specific consent forms were created, incorporating the SS. These were presented to the hospital’s general surgery multidisciplinary team, which consisted of consultants, specialty trainees, foundation doctors, and advanced nurse practitioners. Following feedback from the team, the consent forms were revised to contain the following: (i) a further breakdown of risks, including details to help aid the discussion; (ii) risks grouped by order of prevalence; and (iii) layman's term explanations of what certain risks would result in (e.g., prolonged hospital stay).

The final consent forms were then submitted for use (Supplemental materials 1 and 2). It should be noted that handwritten consent forms were still available for use after the pre-filled consent forms were distributed.

Cycle 2 evaluated the impact of these pre-filled consent forms. A second retrospective analysis was performed after six months. Seventy-four consent forms were analyzed according to the initial retrospective study methodology, and the results between pre- and post-consent form implementation were compared.

This manuscript was prepared in accordance with the SQUIRE 2.0 Standards for Quality Improvement Reporting Excellence guidelines to ensure comprehensive and transparent reporting of the quality improvement project (Supplemental material 3) [21].

Approval statement

This quality improvement study was done in accordance with the UK Health Research Authority (HRA) guidelines. The HRA research criteria determine this study as a service evaluation; therefore, NHS Research Ethics Committee (REC) was not required. This quality improvement study was approved according to the local Trust Audit and Quality Improvement Policy. All participant data was anonymized during data collection.

Data analysis

Statistical significance was calculated using an unpaired T-test statistical analysis. 

## Results

Cycle 1 

Pre-intervention laparoscopic appendicectomy handwritten consent forms had a mean of 64.00% (n = 10) of risks documented when compared to SS. The percentage of risks mentioned in each consent form can be seen in Table 1. Bleeding, bruising, infection, and blood clots were the most commonly mentioned risks, whereas infertility and urinary retention were not listed. 

Similarly, pre-intervention, laparoscopic cholecystectomy handwritten consent forms had a mean of 55.79% (n = 10) of risks listed compared to SS. Pain, bleeding, infection, and injury to the bowel were listed in all consent forms, whereas infertility was never mentioned, and hernias were mentioned only once. 

**Table 1:** Percentage of risks documented compared to our SS for each consent form by procedure, pre-intervention (n = 10 consent forms per procedure)

Laparoscopic appendicectomy		Laparoscopic cholecystectomy	
Consent form (n)	% of risks mentioned compared to SS	Consent form (n)	% of risks mentioned compared to SS
1	73.33	1	57.89
2	93.33	2	57.89
3	46.67	3	52.63
4	53.33	4	78.95
5	60.00	5	57.98
6	53.33	6	73.68
7	53.33	7	57.89
8	66.67	8	31.58
9	66.67	9	57.89
10	73.33	10	31.58

Cycle 2

Of the consent forms analyzed in cycle 2, 74 were analyzed, 42 of which were pre-filled consent forms and 32 handwritten.

Cycle 2 demonstrated an increase in the average number of risks documented on consent forms (both handwritten and pre-filled) compared to the SS. The mean percentage increased to 81.82% (n = 45) for laparoscopic appendicectomy and 79.69% (n = 29) for laparoscopic cholecystectomy (Table 2). 

**Table 2:** Mean number of risks documented pre- and post-intervention

	Mean pre-intervention	Mean post-intervention
Laparoscopic appendicectomy	64.00% (n = 10)	81.82% (n = 45)
Laparoscopic cholecystectomy	55.79% (n = 10)	79.69% (n = 29)

When comparing how the mean number of risks differed between handwritten and pre-filled consent forms post-intervention, we noted that our pre-filled consent forms showed a statistically significant improvement in the mean number of risks documented (as a percentage of the SS) compared to their handwritten counterparts (p < 0.05), which were still in circulation post-intervention (Table 3).

**Table 3:** Comparison of mean number of risks documented per consent form type post-intervention

	Mean post-intervention: handwritten consent forms	Mean post-intervention: pre-filled consent forms	p-value
Laparoscopic appendicectomy	59.10% (n = 20)	100% (n = 25)	<0.05
Laparoscopic cholecystectomy	50.12% (n = 12)	100% (n = 17)	<0.05

## Discussion

This quality improvement study aimed to improve incomplete risk documentation, illegibility, and inconsistency in the information provided to patients during the consenting process. These issues are well-documented in previous literature [1-3,8,12].

Prior to the introduction of the standardized pre-filled consent forms, handwritten consent forms for both procedures demonstrated a lack of comprehensiveness. For laparoscopic appendicectomy, an average of 64.00% (n = 10) of risks were documented, while laparoscopic cholecystectomy showed an even lower average of 55.79% (n = 10). These figures reflect the well-known limitations of handwritten consent forms [1-3,5]. This lack of appropriate documentation may also reflect the human factors clinicians face when consenting for emergency surgeries, such as time pressures and stress.

The introduction of pre-filled consent forms led to marked improvements in documentation. Post-intervention data showed an increase in the mean percentage of risks documented, with laparoscopic appendicectomy reaching 81.82% (n = 45) and laparoscopic cholecystectomy reaching 79.69% (n = 29). It is of note that the mean number of risks of handwritten consent forms did not improve over this time period; as such, any improvements in the mean number of documented risks are due to the pre-filled consent forms. This is further evidenced by our post-intervention analysis, which showed that pre-filled consent forms had a statistically significant increase (p < 0.05) in the mean number of risks documented compared to their handwritten counterparts. These findings reflect previous literature that pre-filled consent forms improve the documentation of risks during the consenting process [3,6,8].

An important goal of this project was to improve communication between surgeons and patients by using the consent forms as a structured guide for the discussion. The designed consent forms aimed to facilitate this by creating a framework for discussion; this being achieved through categorizing risks by prevalence and including layman’s terms for explanations, improving patient engagement and understanding. Additionally, by including layman's terms, patients could later refer back to their copy of the consent form, thus providing another way to understand and retain this information. This is supported by literature, suggesting that procedure-specific consent forms are better tools for ensuring informed consent, as they provide a clear framework for the discussion [6,9]. The consent forms created in this study also gave space for surgeons to list any further complications they deemed relevant, which may pertain to the specific healthcare needs of the patient in question, allowing the consent forms to not only be procedure-specific but patient-specific.

It is also important to emphasize that the use of these consent forms does not replace the individual risk assessment for the patient, nor the need for a comprehensive discussion between the patient and surgeon. Future efforts might focus on developing a teaching session on consent discussions, this dual approach having been shown as being successful in the past [2,7]. These teaching sessions would involve training healthcare professionals in how to have a thorough consenting discussion and how to optimally use the pre-filled consent forms, ensuring that they adapt them to each patient.

Despite the improvements, the use of pre-filled consent forms is not without limitations. As noted in both this study and existing literature, the balance between standardization and the personalization needed for truly informed consent can be challenging [11,12]. The pre-filled forms risk becoming overly prescriptive, potentially encouraging a one-size-fits-all approach that would undermine the principles of informed consent. Additionally, this quality improvement study did not collect qualitative or quantitative data on patient comprehension or satisfaction. While previous studies have shown that pre-filled consent forms improve patient understanding and provide a framework for the consenting discussion, thereby supporting this study’s conclusions, future efforts should include evaluation of both patient satisfaction and comprehension [6,9].

It is also important to note the retrospective nature of this study, as well as the lack of information regarding clinician variability and representation across different grades. These factors may have introduced variability and bias into the analysis. Future efforts may include a prospective study design with appropriate representation across clinician grades and the use of a standardized consenting protocol. Finally, the sample size in Cycle 1 was small (n = 20), and data on who completed the handwritten consent forms was not obtained, which may further contribute to bias. Nevertheless, our findings of the presence of risk omissions and the subsequent improvement in risk documentation are supported by evidence in previous studies [3,5,6,9].

The improvement in consent form documentation not only enhances patient care but also has important legal and professional implications. Informed consent is a legal requirement, and failure to adequately inform patients of potential risks opens up healthcare providers to litigation. The findings of this study, showing an improvement in documented risks, suggest that pre-filled consent forms could help protect surgeons and hospitals from legal challenges. Moreover, the improved documentation process likely contributes to increased patient confidence in their surgical care. Patients who feel that their surgeon has taken the time to thoroughly explain the risks and benefits of a procedure are more likely to trust in the care they are receiving. 

This study underscores the value of pre-filled consent forms in standardizing and improving the informed consent process, particularly in emergency surgical settings where the acuity of patients and the stressors of an on-call shift may contribute to increasing human error. Future steps include (i) a larger sample size to ascertain statistical significance; (ii) devising and implementing teaching sessions surrounding consenting patients and how to correctly use these pre-filled consent forms as an aid; and (iii) it may be of use to use a prospective study design that also includes data collection on patient satisfaction and comprehension. 

Recommendations

Based on the quality improvement project undertaken, a set of recommendations was made to help guide future clinicians in creating effective consent forms. (i) Consent forms should incorporate detailed information to support comprehensive discussions and have risks organized by prevalence. (ii) Layman's terms should be included in consent forms explaining clearly possible outcomes so that patients may refer back to their copy of the consent form for information. (iii) Forms should also allow space for patient-specific risks, ensuring customization and enhancing the validity of the informed consent process. (iv) Thought should be made on how these can then be adapted to a paperless system.

## Conclusions

This quality improvement project has demonstrated the clear benefits of pre-filled emergency surgery consent forms. It shows improvement in the documentation of risks for laparoscopic appendicectomy and laparoscopic cholecystectomy. The forms serve as an effective tool for reducing omissions, variability in risk documentation, and human factors such as illegibility, aligning with the primary objective of this quality improvement study. Additionally, they may help facilitate patient-surgeon communication by acting as a framework for thorough discussion of risks in high-pressure environments. Although this study did not directly measure patient comprehension, the structure and content of the forms are supported by existing literature as tools for improving communication.

Finally, a set of recommendations was developed for the future development of consent forms, which is applicable to a wide variety of procedures. These include (i) recommendations on layman's terms to enhance communication and patient retention, (ii) customization of consent forms to ensure patient-specific consent, and (iii) transitions to electronic consent forms, improving sustainability practices. 

## Figures and Tables

**Figure 1:**
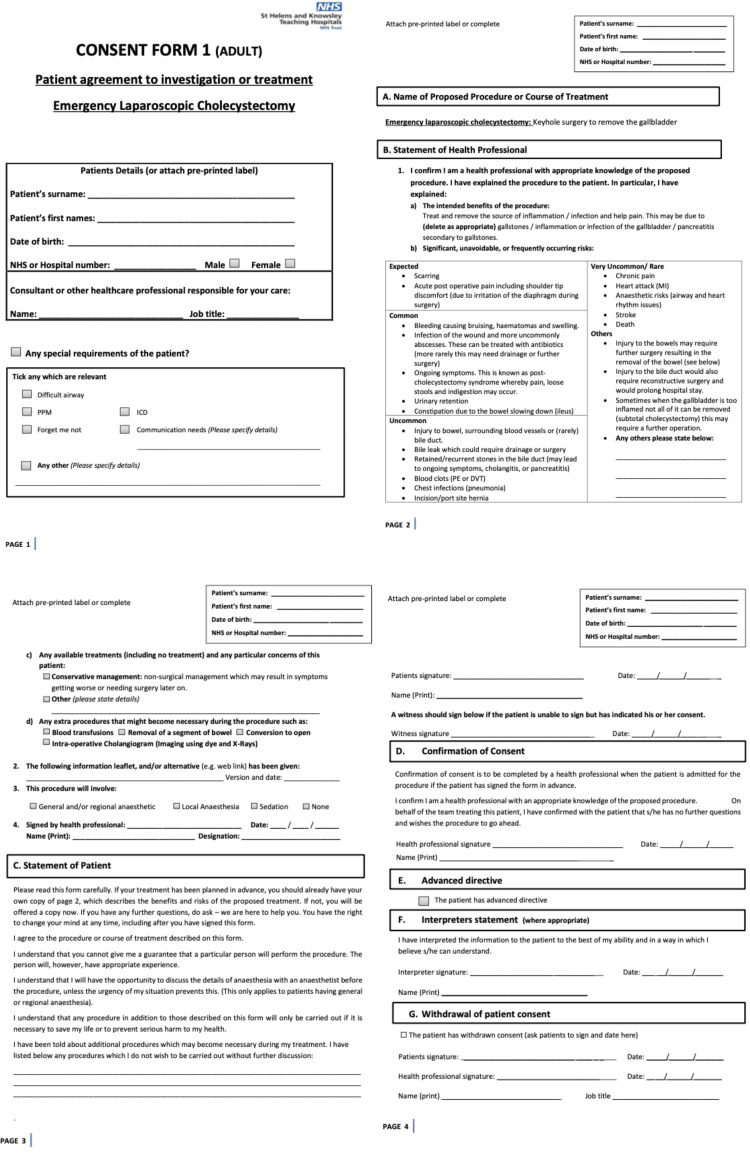
Laparoscopic cholecystectomy consent form

**Figure 2:**
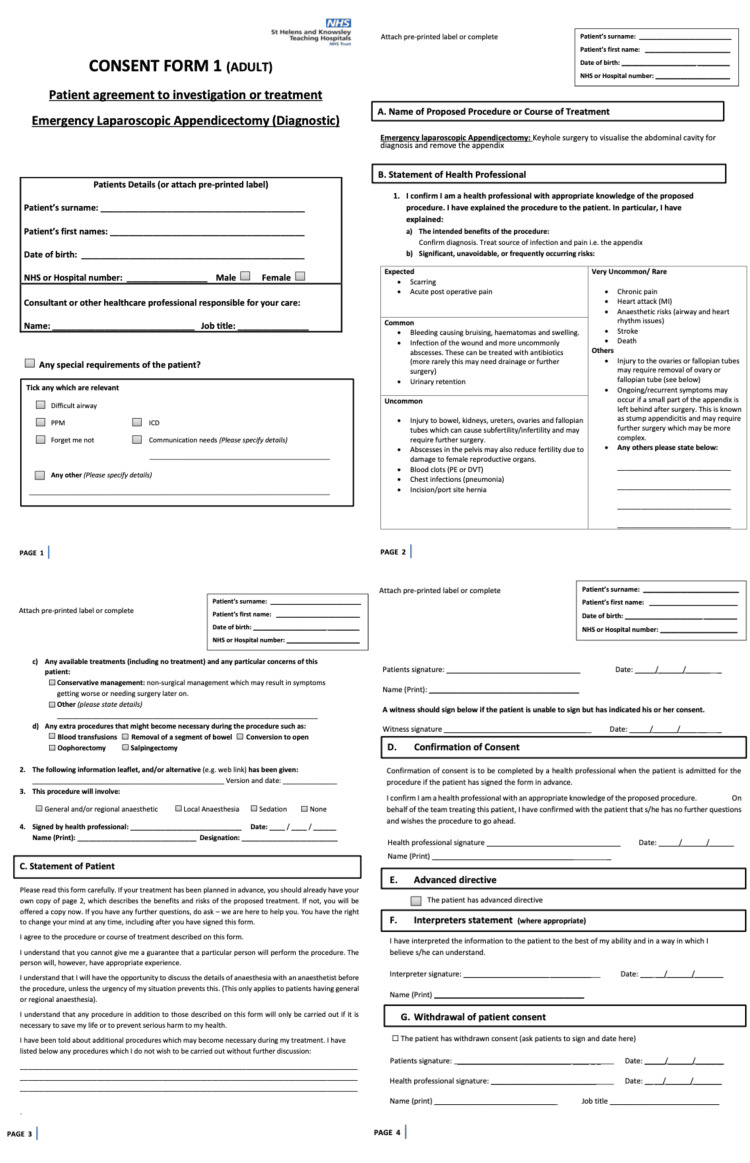
Laparoscopic appendicectomy consent form

**Figure 3:**
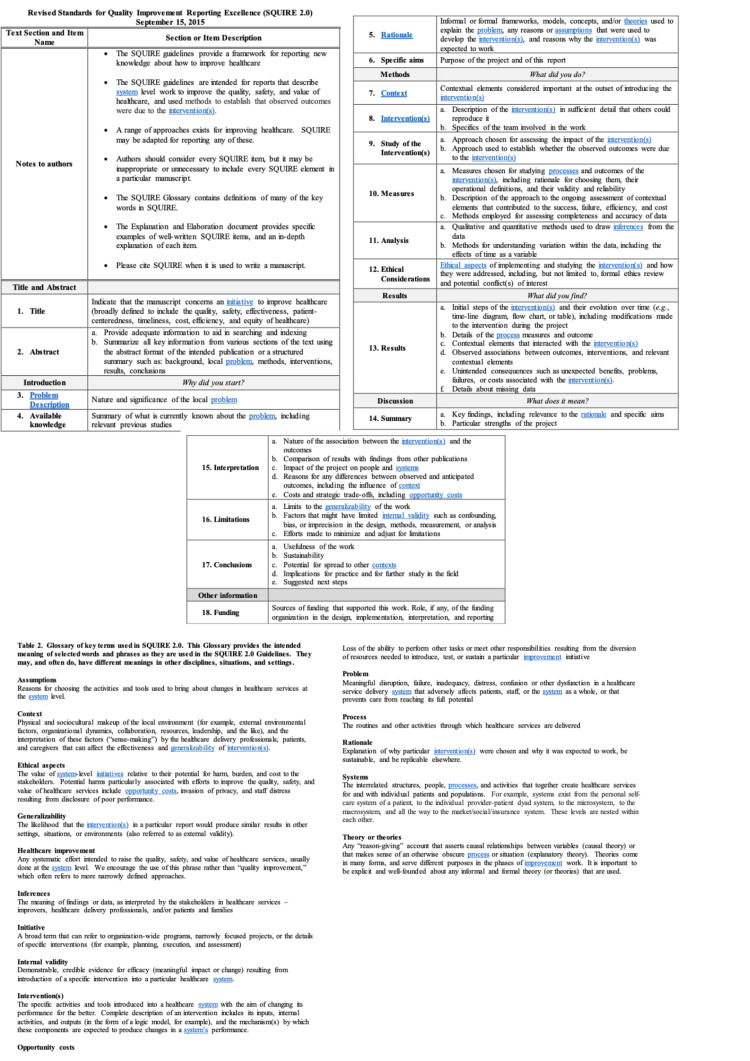
SQUIRE 2.0 checklist

## Appendices

Supplemental material 1 

Supplemental material 2 

Supplemental material 3 
